# Student Perspectives on Aging, Dementia, and Health Care: Lessons Learned from a Community Based Mentorship Program

**DOI:** 10.1093/geroni/igaf122.3659

**Published:** 2025-12-31

**Authors:** Rachel Abramson, Mengru Wang, Marigrace Becker, Stephanie Adaniya, Michael Rosenbloom

**Affiliations:** University of Washington, Seattle, Washington, United States; University of Washington, Seattle, Washington, United States; UW Medicine, Seattle, Washington, United States; University of Washington, Seattle, Washington, United States; University of Washington, Seattle, Washington, United States

## Abstract

Health professional students have limited exposure during their training to the patient and caregiver perspective of aging and living with dementia. Prior studies have shown that community-based service-learning experiences enhance empathy among health professional students. There is limited understanding of how these programs can impact health professional student perspectives on aging, dementia, and the health care system. Ten older adults with mild stage dementia and their caregivers were recruited from a community center that hosts dementia friendly programs. Seven medical students and three social work students at the University of Washington participating in a service-learning program were matched with an older adult mentor and their caregiver based on common interests. Students met with their older adult mentor and caregiver monthly over the course of one year, with activities ranging from walks and attending musical performances, to observing medical appointments. Eight students participated in semi-structured interviews at the conclusion of the program. A thematic analysis was performed by two reviewers using an inductive and deductive approach. Themes identified from the qualitative analysis included appreciating the uniqueness of each person’s experience with dementia, the significant stigma surrounding dementia and aging, barriers to accessing care (technology, transportation, prolonged waiting times to seeing providers), and the importance of community for aging and living well with dementia. Next steps in the study include a qualitative analysis of the older adult mentor and caregiver experiences, and assessment of self-reported quality of life among older adults and perceived caregiver burden pre and post program.

